# Supplementary material for: Empirical methods for controlling false positives and estimating confidence in ChIP-Seq peaks
Source: BMC Bioinformatics. 2008 Dec 5;9:523. doi: 10.1186/1471-2105-9-523 (PMC2628906; doi:10.1186/1471-2105-9-523)
Supplement: Additional File 1 — A variety of html documents from the USeq web site detailing the available applications, their best usage, output file type descriptions, command line menus, etc. [file 1471-2105-9-523-S1.zip › USeqUserGuides/JavaDoc.zip!/JavaDoc/trans/main/ScoredGene.html]

ScoredGene


|  |  |  |
| --- | --- | --- |
| **Overview** \| **Package** \| **Class** \| **Use** \| **Tree** \| **Deprecated** \| **Index** \| **Help** | |  |
| **PREV CLASS**   **NEXT CLASS** | **FRAMES**    **NO FRAMES**     **All Classes** |  |
| SUMMARY: NESTED \| FIELD \| CONSTR \| METHOD | DETAIL: FIELD \| CONSTR \| METHOD |  |


---


## trans.main Class ScoredGene

```
java.lang.Object
  trans.main.ScoredGene
```

**All Implemented Interfaces:**: java.lang.Comparable

---

``` public class ScoredGene extends java.lang.Object implements java.lang.Comparable ```

---

**Constructor Summary**

`ScoredGene(UCSCGeneLine geneReference,
float[][] treatmentValues,
float[][] controlValues)`


| **Method Summary** | |
| --- | --- |
| `int` | `compareTo(java.lang.Object obj)` |
| `float[][]` | `getControlValues()` |
| `UCSCGeneLine` | `getGene()` |
| `double` | `getMedian()` |
| `double` | `getMisc()` |
| `float[][]` | `getTreatmentValues()` |
| `void` | `setMedian(double median)` |
| `void` | `setMisc(double misc)` |

**Methods inherited from class java.lang.Object**

`equals, getClass, hashCode, notify, notifyAll, toString, wait, wait, wait`

**Constructor Detail**

### ScoredGene

```
public ScoredGene(UCSCGeneLine geneReference,
                  float[][] treatmentValues,
                  float[][] controlValues)
```


**Method Detail**

### compareTo

```
public int compareTo(java.lang.Object obj)
```

:   **Specified by:**: `compareTo` in interface `java.lang.Comparable`

---


### getMedian

```
public double getMedian()
```

---


### setMedian

```
public void setMedian(double median)
```

---


### getControlValues

```
public float[][] getControlValues()
```

---


### getGene

```
public UCSCGeneLine getGene()
```

---


### getTreatmentValues

```
public float[][] getTreatmentValues()
```

---


### getMisc

```
public double getMisc()
```

---


### setMisc

```
public void setMisc(double misc)
```


---


|  |  |  |
| --- | --- | --- |
| **Overview** \| **Package** \| **Class** \| **Use** \| **Tree** \| **Deprecated** \| **Index** \| **Help** | |  |
| **PREV CLASS**   **NEXT CLASS** | **FRAMES**    **NO FRAMES**     **All Classes** |  |
| SUMMARY: NESTED \| FIELD \| CONSTR \| METHOD | DETAIL: FIELD \| CONSTR \| METHOD |  |


---
